# On the Perplexingly Low Rate of Transport of IgG2 across the Human Placenta

**DOI:** 10.1371/journal.pone.0108319

**Published:** 2014-09-24

**Authors:** Helga K. Einarsdottir, Nigel M. Stapleton, Sicco Scherjon, Jan Terje Andersen, Theo Rispens, C. Ellen van der Schoot, Gestur Vidarsson

**Affiliations:** 1 Department of Experimental Immunohematology, Sanquin Research and Landsteiner Laboratory, Academic Medical Centre, University of Amsterdam, Amsterdam, The Netherlands; 2 Department of Obstetrics and Gynacology, University Medical Centre, Groningen, The Netherlands; 3 Centre for Immune Regulation (CIR) and Oslo University Hospital, Department of Immunology, Oslo, Norway; 4 Department of Immunopathology, Sanquin Research and Landsteiner Laboratory, Academic Medical Centre, University of Amsterdam, Amsterdam, The Netherlands; Institut National de la Santé et de la Recherche Médicale U 872, France

## Abstract

The neonatal receptor, FcRn, mediates both serum half–life extension as well as active transport of maternal IgG to the fetus during pregnancy. Therefore, transport efficiency and half-life go hand-in-hand. However, while the half-life of the human IgG2 subclass is comparable to IgG1, the placental transport of IgG2 is not, with the neonatal IgG1 levels generally exceeding maternal levels at birth, but not for IgG2. We hypothesized that the unique short-hinged structure of IgG2, which enables its κ-, but not λ-isotype to form at least three different structural isoforms, might be a contributing factor to these differences. To investigate whether there was any preference for either light chain, we measured placental transport of IgG subclasses as well as κ/λ-light chain isotypes of IgG1 and IgG2 in 27 matched mother-child pairs. We also studied the half-life of IgG1 and IgG2 light chain isotypes in mice, as well as that of synthesized IgG2 structural isotypes κA and κB. In order to investigate serum clearance of IgG1 and IgG2 light-chain isotypes in humans, we quantified the relative proportions of IgG1 and IgG2 light chains in hypogammaglobulinemia patients four weeks after IVIg infusion and compared to the original IVIg isotype composition. None of our results indicate any light chain preference in either of the FcRn mediated mechanisms; half-life extension or maternal transport.

## Introduction

Immunoglobulin G (IgG) forms the backbone of our circulating, adaptive immune system. The fully assembled IgG molecule consists of two identical 50 kDa heavy chains (γ1, γ2, γ3 or γ4 subclasses), and two identical 23 kDa light chains forming a heterodimer (one heavy chain and one light chain) that further assemble into dimers. The assembled molecule is Y shaped, with the light chains and the N-terminal parts of the heavy chains (C_H_1 and V_H_ domains) in tight association, forming the two Fab arms (Fragment antigen binding), and the C-terminal C_H_2 and C_H_3 domains forming the Fc-tail. The C_H_1 and C_H_2 domains are connected by a flexible hinge, allowing the F(ab′)_2_ considerable freedom of movement from the Fc portion. Length and flexibility of the hinge region varies extensively amongst the IgG subclasses influencing the relative orientation and movement of the Fab arms and Fc tail of the IgG antibody [1].

The hinge region of IgG1 encompasses 15 amino acids and is very flexible. IgG2 has a 12 amino acid hinge region and contains a rigid poly-proline double helix, stabilized by four inter-heavy chain disulfide bridges. IgG3 has the longest hinge region, about 4 times as long as IgG1, and thus the greatest flexibility, while the IgG4 hinge contains 12 amino acids yielding an intermediate flexibility compared to IgG1 and IgG2 [2].

Light chains come in two classes, either κ or λ, with four highly homologous λ light chain allotypes. In humans the κ∶λ ratio in serum is around 2∶1 in healthy individuals, but this varies between species, isotype, biological location and age [3]. No functional differences between λ and κ antibodies have been described so far. Recently, IgG2κ was described to occur in three distinct isoforms, A, A/B and B, which differ from each other solely in their disulphide bridges in the hinge, with four disulphide bonds connecting the Fc chains for A, but two in the B form, and an hybrid A/B form with three inter-Fc bonds [4]. This affects its tertiary structure and thus the position and mobility of F(ab′)_2_, which in turn may affect other interactions [5]. In contrast, IgG2λ is found predominantly as the A and A/B molecular form, but devoid of the B form [4], [6].

FcRn, the neonatal FcγR, is a heterodimer of a unique MHC class I-like alpha chain and β_2_m. It is thought to, amongst other functions, be responsible for both the long half-life of IgG *in vivo* and to mediate IgG transcytosis, for instance to the mucosa (e.g. gut, genital and respiratory tract) and through the placenta [7]–[10]. FcRn functions by binding IgG in acidifying early pino- or endosomes after these have fused with FcRn bearing vesicles [11]. Once bound, IgG-FcRn complexes are routed away from the lysosomal pathway, either back towards the loading surface of the cell (recycling) or to the opposite cell surface (transcytosis). After fusion with the cell membrane the pH returns to its physiological value and the IgG-FcRn complexes dissociate, allowing the IgG to disperse outside of the cell [12]. In humans, FcRn thereby prolongs the half-life of all subclasses equally, except for IgG3. We recently demonstrated that this short half-life of IgG3 was caused by alteration in a key FcRn-contact residue in IgG3 compared to the other subclasses (where IgG3 has an Arginine at position 435 instead of Histidine). This causes IgG3, in most individuals, to have less pH sensitive binding to FcRn and therefore to lose in competition with other subclasses, except for those expressing a naturally occurring IgG3 variant (G3m15 and G3m16) with Histidine at 435 (H435) [13].

IgG1, IgG2 and IgG4 share all currently described contact residues with FcRn, including H435, with similar affinity to FcRn when measured using immobilized human FcRn on a biosensor and a comparable half-life in circulation [14]–[16]. For transport of IgG across the placenta however, the concentration of all the H435-containing IgG's in cord blood except IgG2 (i.e. IgG1, IgG4 and H435-containing allotypes of IgG3) typically exceeds maternal levels. Thus the transport of IgG2 is much less efficient and is in fact comparable to that of R435-containing IgG3 [17], [18]. Since FcRn is thought to play a very similar key role in both of these processes, the reason for this discrepancy remains unclear.

Interestingly, although all amino acids so far found to influence FcRn binding are reported to be located on the Fc portion of IgG, Montaño and Morrison reported a difference in half-life between human-mouse chimeras of IgG2λ and IgG2κ, with IgG2λ being cleared faster in mice [15], [19]. This light-chain difference was not observed for IgG1, IgG3 or IgG4. All human IgG subclasses have been reported to display pH-dependent binding activities to mouse FcRn, albeit slightly altered compared to binding to human FcRn [16]. The interaction still allows for half-life extension of human IgG by mouse FcRn, with comparable relative half-lives reported as in humans (IgG1≥IgG2≫IgG3), except for IgG4, which has been reported to have unusually short half-life in mice [15], [19]–[22]. Although no difference in IgG2 half-life has been described in humans, the IgG2 light chain isotype has been found to influence binding affinity to mouse FcRn [23]. Due to the shortness of the IgG2 hinge, which causes the F(ab)_2_ to be relatively close to the Fc as well as the distinct structural isoforms of IgG2, we hypothesized that the low placental transport of IgG2 may be attributable to a single structural isoform, unique to IgG2κ, interacting differently with FcRn, possibly affecting its half-life as well.

For this reason we studied the trans-placental transport of IgG2λ and IgG2κ in humans and their half-life in mice and humans, both of which are FcRn-ascribed functions, and tested whether the unique structural isoforms of IgG2κ could explain the low efficiency of IgG2 transport.

## Materials and Methods

### Recombinant IgG

V_H_-matched recombinant IgG1 (λ and κ) and IgG2 (λ and κ) described before [24], [25], were produced in 293 Freestyle cells (Invitrogen, Carlsbad CA) according to the manufacturer's instructions with p20, p27 and SV40 Large-T antigen as described, and purified by protein G HiTrap columns using the Acta Prime Plus system (GE Healthcare, Buckinghamshire, UK) [26].

### Paired Mother – Cord samples

IgG subclass levels and for both IgG1 and IgG2 the kappa and lambda levels, were measured in plasma samples from paired mothers and cord, all at term, taken just before or just after birth. Median gestation period was 268 (range 281 and 252 days), except otherwise indicated. The data was plotted for each mother/child combination individually and also assessed collectively. All women had an uncomplicated pregnancy and neonatal outcomes for all children were optimal. Signed informed consent was obtained from all women, and the collection of blood samples and clinical data received approval by the Ethics Committee of the Leiden University Medical Center (P02-200).

### IgG quantification

Light chain and IgG1 and IgG2 subclass specific antibody quantification on the paired mother-cord samples was done by sandwich ELISA using Nunc MaxiSorp plates (Sigma-Aldrich, St. Louis, MO). For IgG total ELISA, mouse anti-human IgG (M1268, Sanquin) was used for coating (1/500), and HRP-mouse anti-human Fc (JDC10 Southern Biotech) was used as a secondary antibody (1/1000). For IgG light chain ELISA's mouse-anti-human IgG1 (MH1325, Sanquin, 1/100) was used to coat, using either HRP-labelled mouse-anti-human λ (JDC12, Southern Biotech, 1/1600) or κ (HP6062, Southern Biotech, 1/1300). Similarly, mouse anti-human IgG2 (HP6002, Southern Biotech, 1/100 for lambda assay, 1/50 for kappa assay) was used for coating, and HRP-labelled mouse anti-human λ JDC12 (Southern Biotech, 1/1600) or k (HP6062, 1/1300) for detection.

Conversion of 3,3′,5,5′-Tetramethylbenzidine (TMB) was used to quantitate HRP activity per well and absorptions were read using a Genios Pro plate reader (Tecan, Männedorf, Switzerland) using standard sets of filters of 450 nm.

IgG subclass concentrations in sera were determined by nephelometry (Behringer nephelometer II, Behringer diagnostics).

### *In vivo* serum persistence experiments

BALB/c mice were obtained from the NKI institute in Amsterdam. C57Bl/6 and C57Bl/6-FcRn-KO mice were obtained from the Charles River and Jackson laboratories, respectively. All *in vivo* experiments were performed using female 8–9 week old mice weighing between 16.8 and 26.3 grams each. Each mouse received 200 µg purified recombinant human IgG of the appropriate heavy and light chain subclass and isoform by IV administration. Blood samples of 100 µl were drawn from the tail at day 0 and then approximately every 5 days for a period of two weeks, and human IgG concentration was determined with a mouse anti-human IgG ELISA. All animal experiments were carried out after approval from the ethical committee of Sanquin/Dutch Cancer Institute (Dierexperimentencommissie NKI), who also monitored the progress, aimed at minimizing suffering (number 10.033).

In humans IgG light chain subclass serum persistence was calculated as (%) = [IgG_S_]_W4_/[IgG_T_]_W4_/([IgG_S_]_IVIg_/[IgG_T_]_IVIg_)×100% in sera from three agammaglobulinemic patients receiving IVIg at 4 weeks interval as described in [20].

### IgG2κ redox treatment

Enrichment of IgG2 κ isoforms by redox treatment was performed as described in Dillon et al 2008 (Structural and Functional Characterization of Disulfide Isoforms of the Human IgG2 Subclass) [5]. Shortly, for the synthesis of the B isoform, IgG2κ was incubated at 3 mg/ml in 200 mM Tris buffer at pH 8 with 6 and 1 mM of cysteine and cystamine, respectively. For IgG2κ A enrichment, 0.9M guanidine hydrochloride (GuHCl) was also added. The samples were protected from light and placed at 2–8°C for 48–72 h. Afterwards the antibody was run through a Zeba spin desalting column (Pierce) for buffer exchange into PBS, and stored at −20°C.

### SDS gel electropheresis

5 µg of protein was loaded on a Thermo Scientific Tris Hepes SDS precast polyacrylamide mini gel 12%. Reduced samples were diluted with NuPAGE LDS Sample Buffer (4×) with 0.1% Beta MercaptoEthanol and incubated at 95C° for 5 minutes. Non- reduced samples were diluted with NuPAGE LDS Sample Buffer (4×) with 60 mM Iodoacetamide and incubated at 70°C for 5 minutes The running buffer was TRIS HEPES SDS buffer for both types of samples, for the non-reduced samples 4 mM Iodoacetamide was added. Run time was ca 1.5 hour at 130 mA/gel, 120 V. Afterwards, gels were stained with Coomassie blue.

### Surface plasmon resonance

Surface plasmon resonance was conducted using a Biacore 3000 instrument (GE Healthcare) with CM5 sensor chips. The coupling was performed by injecting 5 µg/ml of the each protein into 10 mM sodium acetate pH 4.5 using the amine coupling kit (GE Healthcare). Titrated amounts (8,000.0-62.50 nM) of monomeric human FcRn were injected over immobilized IgG variants (500 RU) using phosphate buffer (67 mM phosphate buffer, 0.15 M NaCl, 0.005% Tween 20) at pH 6.0 as running buffer whereas HBS-P buffer at pH 7.4 was used for regeneration of the flow cells. Experiments were conducted at 25°C with a flow rate of 40 µl/min. All sensorgrams were zero adjusted and reference cell values subtracted before analyzed using the BIAevaluation 4.1 software.

### CEX-HPLC Analysis of IgG2κ A and B isoforms

CEX-HPLC analysis was carried out as described previously [4]. Shortly, an ÄKTA Avant system from GE healthcare with a Dionex ProPac WCX-10 (4.0_250 mm) column was used for analysis of recombinant IgG preparations. Mobile phase A was 20 mM sodium acetate, pH 5.0, and mobile phase B was the same with 0.5 M NaCl added. Protein was eluted during a linear gradient from 20% to 80% B at a flow rate of 0.5 ml/min.

## Results

### IgG2 and IgG3 are not efficiently transported across the placenta

**Figure 1:**
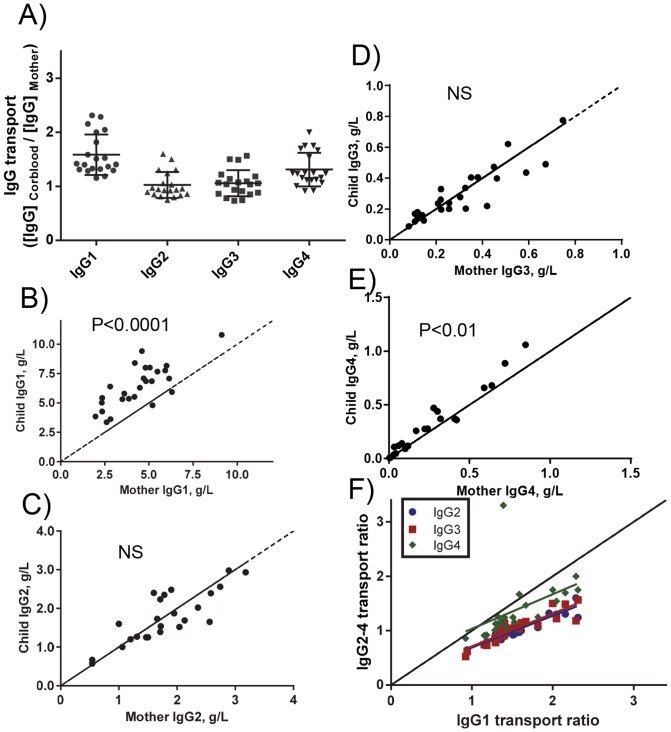
Placental transport of IgG subclasses is more efficient for IgG1 and IgG4 than for IgG2 and IgG3. Blood was collected from mothers just before or after birth and from neonates birth. A) Transport rates for all IgG subclasses expressed as cord/maternal ratios found at birth. The transport rates differed significantly from each other (P<0.0001), except for IgG2 and IgG3 (not significant), as tested by one-way Anova and Tukey's multiple comparison test. (B–E)IgG subclass 1–4 serum levels were quantified by nephelometry and each pair was plotted on a X axis displaying days of each pregnancy against IgG concentration. Average neonate concentration was significantly higher than in the mother for IgG1 and IgG4 as tested by a paired-T test as shown (child/mother ratio = 1.55 and 1.38, respectively) while averge concentrations for IgG2 and IgG3 were not significantly different in mothers and their children (child/mother ratios not significantly different from 1). One pre-term baby was identified displaying low transport of all IgG (square symbol). (F) Child/mother transport ratio of subclasses IgG2-4 for each pair was plotted relative to the IgG1 transport ratios.

We quantified all four IgG subclasses in 27 paired mother cord plasma samples taken at birth (Fig. 1A–D). As expected, IgG1 and IgG4 levels in cord blood were generally found to be higher than in maternal blood. Specifically, cord exceeded maternal levels in 24 out of 27 samples with the average cord/mother ratio being 1.5 for IgG1, 1.3 for IgG4 while for IgG3 the average cord/mother ratio was 1.00. IgG2 levels in cord and maternal plasma were also about equal (with average cord/mother ratio being 0.99). Interestingly, the pairs show a highly varied transport, with some individuals transporting all subclasses at higher efficiency than others. However, the overall transport was relatively uniform for all the subclasses: if relatively high transport was observed for IgG1, the same held true for IgG2 (R^2^ = 0.875, p<0.0001), IgG3 (R^2^ = 0.836, p<0.0001) and IgG4 (R^2^ = 0.303, p = 0.0052) (Fig. 1E). One case with extremely strong IgG4 transport, with maternal levels being very low (0.03 g/L) but rising to 0.11 g/L in the fetal circulation (Fig. 1E). Due to the uncertainty of measurements at this range, this value was excluded from further analysis.

**Figure 2:**
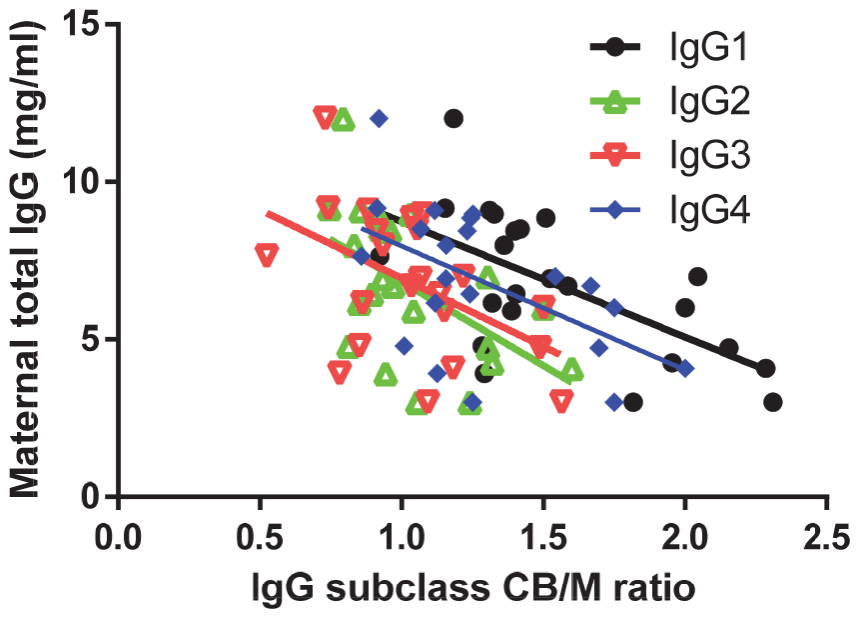
Placental transport efficiency is relatively higher at lower maternal levels. In general, IgG1 is transported better than IgG4, both of which are transported better than IgG2 and IgG3, which have similar transport rates. Two-tailed Pearson correlation revealed a significant correlation for all subclasses, IgG1 R^2^ = 0.379, P = 0.0018; IgG2 R^2^ = 0.2910, P = 0.0096; IgG3 R^2^ = 0.2415, P = 0.0202; IgG4 R^2^ = 0.2881, P = 0.0121. Thus, for all subclasses, relatively more IgG was transported at lower maternal IgG.

In general, the relative transport of all subclasses was in direct relationship to the maternal level: the higher the maternal IgG, the lower the transport observed for all IgG subclasses (Fig. 2). The placental transport rates were IgG1>IgG4>IgG2 = IgG3, at all levels for maternal IgG. The slopes of the respective trend lines were IgG1: −3.664±1.023; IgG2: −5.332±1.861; IgG3: −4.434±1.721; IgG4: −3.963±1.420.

### IgG2κ and IgG2λ are transported equally well over the placenta

**Figure 3:**
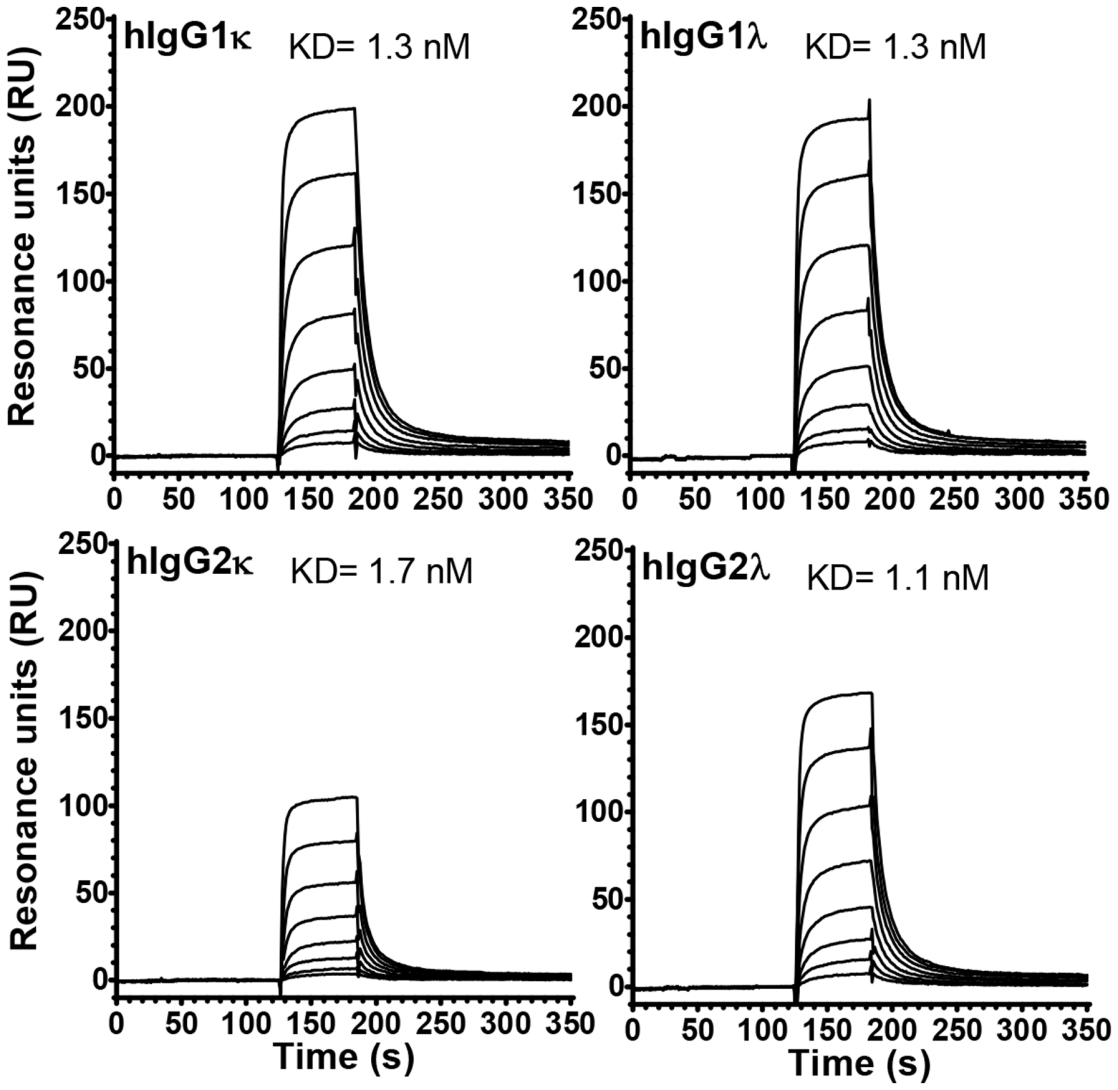
Determination of the FcRn binding properties of IgG1 and IgG2 light chain variants. Binding of titrated amounts of soluble human FcRn (62.5–8000 nM) at pH 6.0 to human IgG variants immobilized onto CM5 sensor flow cells. The relative affinity constants derived (KD) are indicated.

Montano and Morrison described that FcRn-mediated half-life of human-mouse chimeric IgG2λ was shorter than IgG2κ in Balb/c mice, while the FcRn-mediated half-life of light chain isotype of the other subclasses was unaffected [15]. Later the same group described increased affinity of mouse FcRn to human IgG2λ compared to IgG2κ [23]. We therefore investigated if this was also true for human FcRn by measuring the affinity of soluble monomeric human FcRn to immobilized IgG variants at pH 6.0 using surface plasmon resonance. We found FcRn to bind IgG2λ slightly stronger than IgG2κ (KD = 1.1 µM and 1.7 µM, respectively) (Fig. 3). However, no difference in binding affinity was detected for the IgG1-light chain isotypes (KD = 1.3 µM).

**Figure 4:**
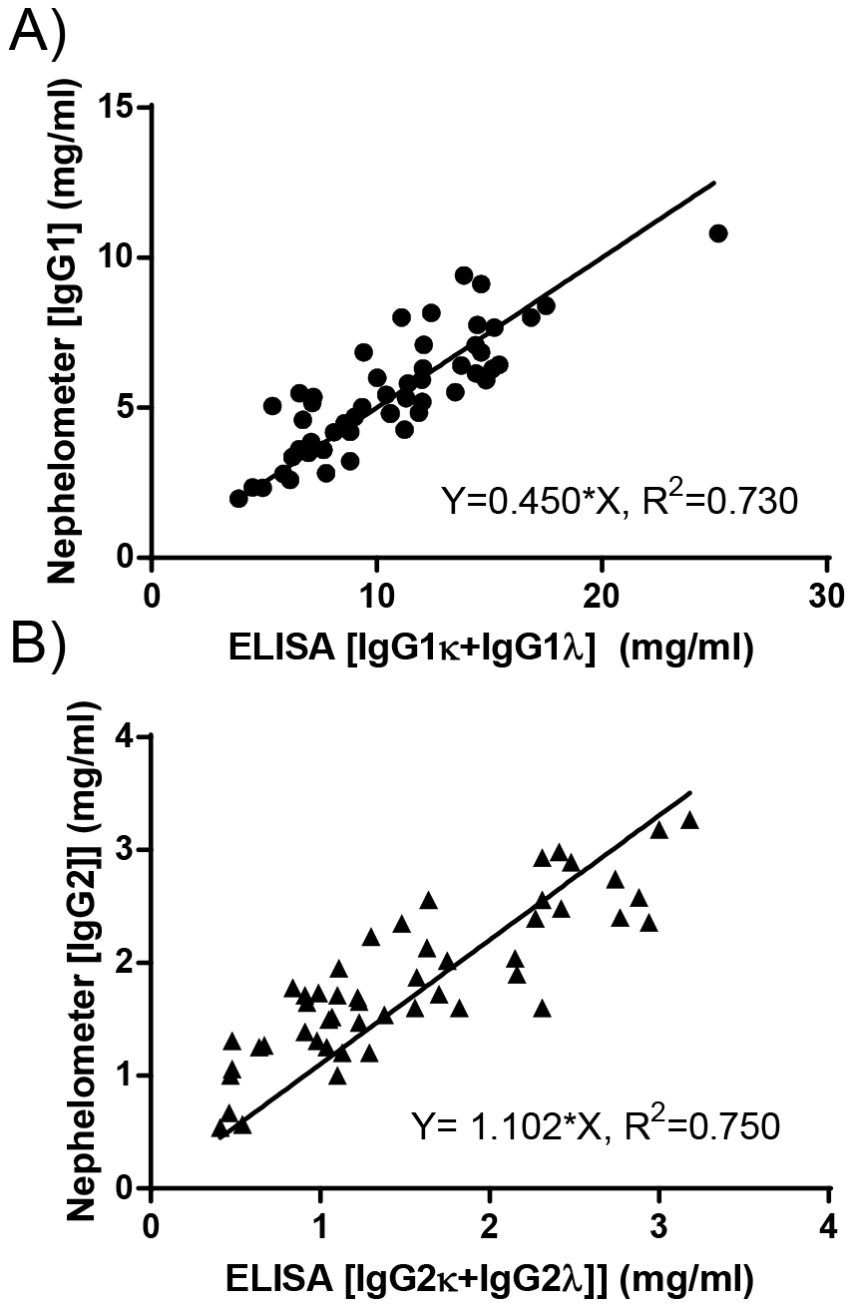
Validation of IgG1- and IgG2- light chain specific ELISA. Results from IgG1 total (A) and IgG2 total (B) were plotted against the sum of IgG1κ and IgG1λ, or IgG2κ and IgG2λ, respectively. The results of regression analysis are indicated in each panel, along with Pearson's correlation.

Although somewhat counterintuitive, these findings raised the possibility that placental transport of IgG2κ and λ may also differ as both half-life and placental transport is mediated by FcRn. To test this, we first set up light chain-specific IgG1 and IgG2 ELISA's for quantification of IgG1κ, IgG1λ, IgG2κ and IgG2λ using recombinant IgG molecules as standards. The light-chain subclass ELISA were validated against the nephelometry data, which correlated well with the sum obtained from the ELISA as determined by regression analysis (Figure 4). Thus, nephelometry data fitted very well for IgG2 quantitatively (slope 1.10), but for IgG1 the nephelometry data seemed to be somewhat underestimated (slope 0.45), or overestimated by the ELISA (or both). In support of this, the average κ/λ ratios were 1.4±0.4 (range 0.7–2.3) and 2.4±1.0 (range 0.8–4.5) for IgG1 and IgG2, respectively, suggesting IgG1λ values to be overestimated by the ELISA as total IgG κ/λ ratios have been estimated to be around 2.0, which has never been determined for the IgG subclasses to our knowledge [27]. Importantly, the sum of the light chain ELISA values for either IgG1 or IgG2 κ and λ correlated equally well with nephelometry data (Figure 4, R**^2^** = 0.73 and 0.75 for IgG1 and IgG2 respectively, p<0.0001). Thus, we concluded that the inconsistency seen between the estimated levels for IgG1 by nephelometry and IgG1κ+IgG1λ was constant over the whole concentration range, suggesting the possible skewing to be relative, and not affecting the relative IgG1κ and IgG1λ ratios measured at either high or low IgG1 levels.

**Figure 5:**
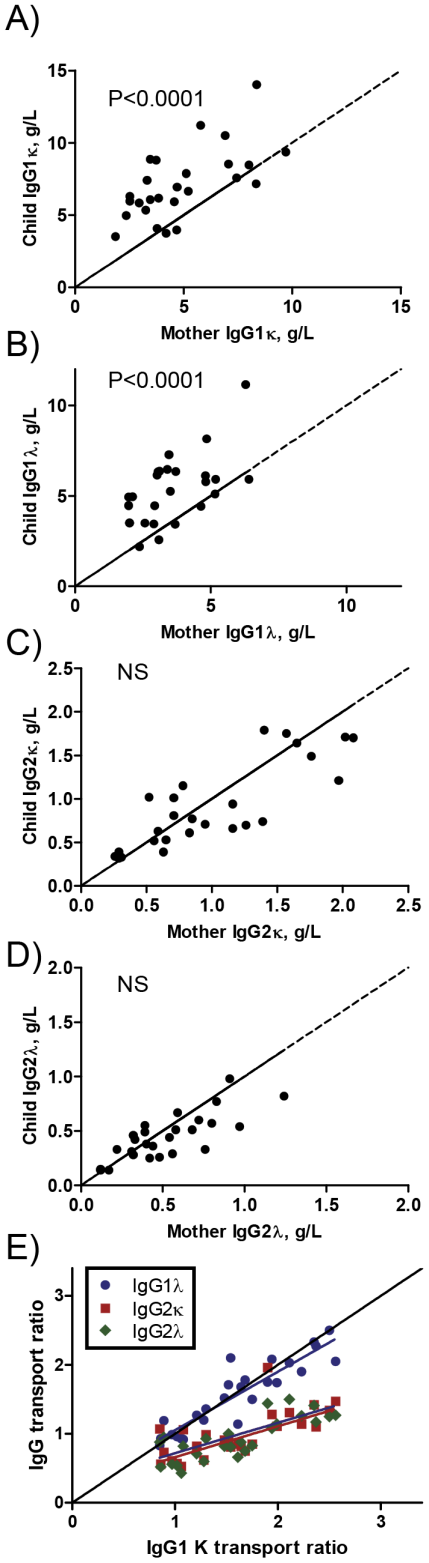
Equal placental transport of κ and λ of IgG1 and IgG2 subclasses. IgG1κ (A), IgG1λ(B) and IgG2κ (C) IgG2λ (D) light chain isotype from sera in Fig. 1 were quantified by subclass- and light chain specific ELISA and each mother-child pair was plotted on the x- and y-axis, respectively. A paired t-test revealed no significant difference between the light chains isotypes within each antibody subclass. Average neonate concentration was significantly higher than in the mother for IgG1κ (A) and IgG1λ (B) as indicated in each graph by P values (child/mother ratio = 1.60 and 1.56, respectively) while average concentrations for IgG2 κ and IgG2 λ (C and D) were not significantly different in mothers and their children. (E) Child/mother transport ratio of IgG1λ, IgG2κ and IgG2λ for each pair was plotted relative to IgG1κ transport ratios. While both IgG2 isotypes perform worse than IgG1 when concentration increases, no difference is visible between the IgG2-light chain isotypes.

Using this method, we estimated that the transport of both IgG1κ and IgG1λ was comparable with a cord/mother ratio of 1.6 (Fig. 4A–B). Placental transport of IgG2 was also equally efficient for both light chain variants (average IgG2 cord/mother ratio = 0.99 and 0.93 for κ and λ, respectively) (Fig. 5C–D). Again, the relative transport for the light chain isotypes (Fig. 5E) was comparable as it was for their respective IgG subclasses (Fig. 1A). There was a strong correlation between IgG1κ and IgG1λ transport (R^2^ = 0.860. p<0.0001), and a weaker one between IgG1κ and the IgG2 two isotypes (R^2^ = 0.549, p<0.0001 and R^2^ = 0.698, p<0.0001 for κ and λ, respectively) (Fig. 5E). Again, the relative transport for the light chain isotypes (Fig. 5E) was similar as observed for the IgG subclasses (Fig. 1A). The more IgG1κ transported, the more IgG1λ was transported as well, which was also similarly increased, but to a lower level, for both IgG2 light chain isotypes as IgG1κ transport correlated significantly with that of and IgG1λ (R^2^ = 0.860, p<0.0001), IgG2κ (R^2^ = 0.549, p<0.0001), and IgG2λ (R^2^ = 0.698, p<0.0001) (Fig. 5E). No difference was found between the slopes for IgG2κ and IgG2λ. Although the slope for IgG1λ differed significantly form IgG1κ (P = 0.030), the difference was minimal, with slightly lower IgG1λ transport at high IgG1κ and vice versa (Fig. 5E). However, paired comparison between either IgG1 κ and λ or IgG2 κ and λ subclass yielded no significant difference. Also, the relative transport of IgG1κ and IgG1λ, as well as that for IgG2κ and IgG2λwere not significantly different.

### The half-life of IgG2 light chain isoforms does not differ in mice

**Figure 6:**
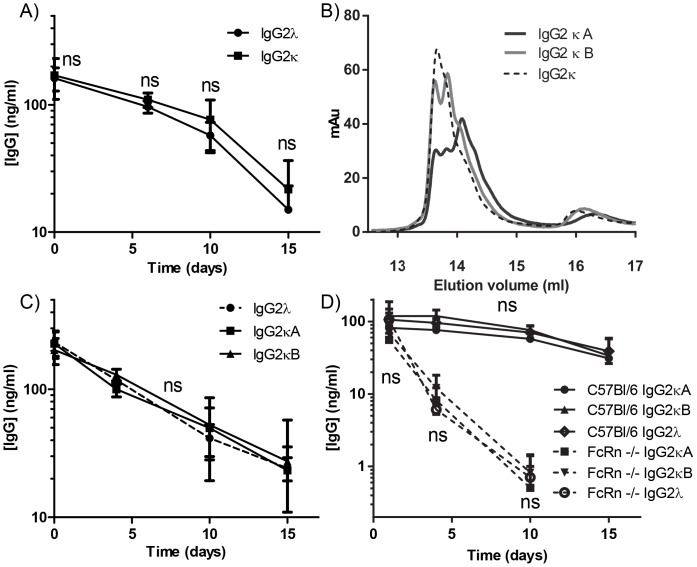
No difference in half-life of IgG2 light chain isotypes κ and λ in mice. (A) Recombinant human IgG2κ and λ in Balb/C mice was injected and measured by total IgG ELISA for a two week period following injection of 200 µg IgG. Calculated half-lives were 7.2±1.48 and 6.4±0.84 days for IgG2κ and IgG2λ. (B) Enrichment of IgG2 κ isoforms was performed as described in Dillon et al 2008. HPLC elution profiles of IgG2 κA and κB structural isomeres on a Dionex ProPac WCX-10 (4.0_250 mm) column are depicted. IgG2κB isoform was generated by incubation of 3 mg/ml IgG2κ in 200 mM Tris buffer at pH 8 with 6 and 1 mM of cysteine and cystamine, respectively. For IgG2κA synthesis 0.9M guanidine hydrochloride (GuHCl) was also added. The samples were kept in the dark and placed at 4°C for 48–72 h. Following incubation the antibody was run on a Zeba spin desalting column (Pierce) for buffer exchange into PBS. (C) The clearance of IgG2λ, IgG2κA, and IgG2κB in Balb/C mice. Calculated half-lives were 4.0±0.58, 5.39±0.85, and 3.7±1.04 days for IgG2κA, IgG2κB and IgG2λ, respectively. (D) Clearance of IgG2κA and IgG2κB in WT and FcγR −/− C57Bl/6 mice. Calculated half-lives were 6.2±2.62, 6.43±1.69, and 7.5±1.89 days for IgG2κA, IgG2κB and IgG2λ, respectively, in WT mice but 1.08±0.28, 1.19±0.23, and 0.7±0.92 days for IgG2κA, IgG2κB and IgG2λ, respectively, in FcRn −/− mice. Graphs in (A, C–D) depict mean and standard deviations of results obtained for 4 mice per group. Half-lives were calculated assuming exponential decay and reported in days ± standard error of means. No significant difference in half-life was detected between the two isotypes.

As a previous study reported a different half-life for IgG2κ and IgG2λ, our next step was to measure the serum half-life of IgG2κ and IgG2λ in Balb/C mice injected with 200 µg of recombinant fully human IgG2κ or IgG2λ [15]. Concentration of human IgG in their serum was quantified at every 5 days over a two week period post injection. No difference was found between the clearance rates of IgG2κ and IgG2λ (Fig. 6A). We then tested if the clearance rate of IgG2κ might be affected by the A/B isoforms recently described [4], [6], [28]. The different isoforms were deliberately generated as described previously (Fig. 6B) [5]. The clearance of neither IgG2κ isoform differed from IgG2λ, nor did the clearance of IgG2κA differ from IgG2κB in Balb/C mice (Fig. 6C). In addition, IgG2κA and IgG2κB were also cleared at equal rates in C57Bl/6 mice (Fig. 6D). C57Bl/6 FcRn KO mice cleared human IgG much faster than both WT expressing mice and displayed no difference between the two IgG2κ isoforms (Fig. 6D). Antibody half-life was determined to be around 10 days in C57Bl/6 mice and around 4 days in Balb/C mice. In the FcRn KO mice, the levels had dropped below the detection limit of the assay at day 10. In conclusion, neither the half-life nor the transplacental transport is affected by the light chain isotype of IgG2 and thus not on structural isomerization of IgG2.

### IgG2 half-life is identical between the light chain isotypes in humans

**Figure 7:**
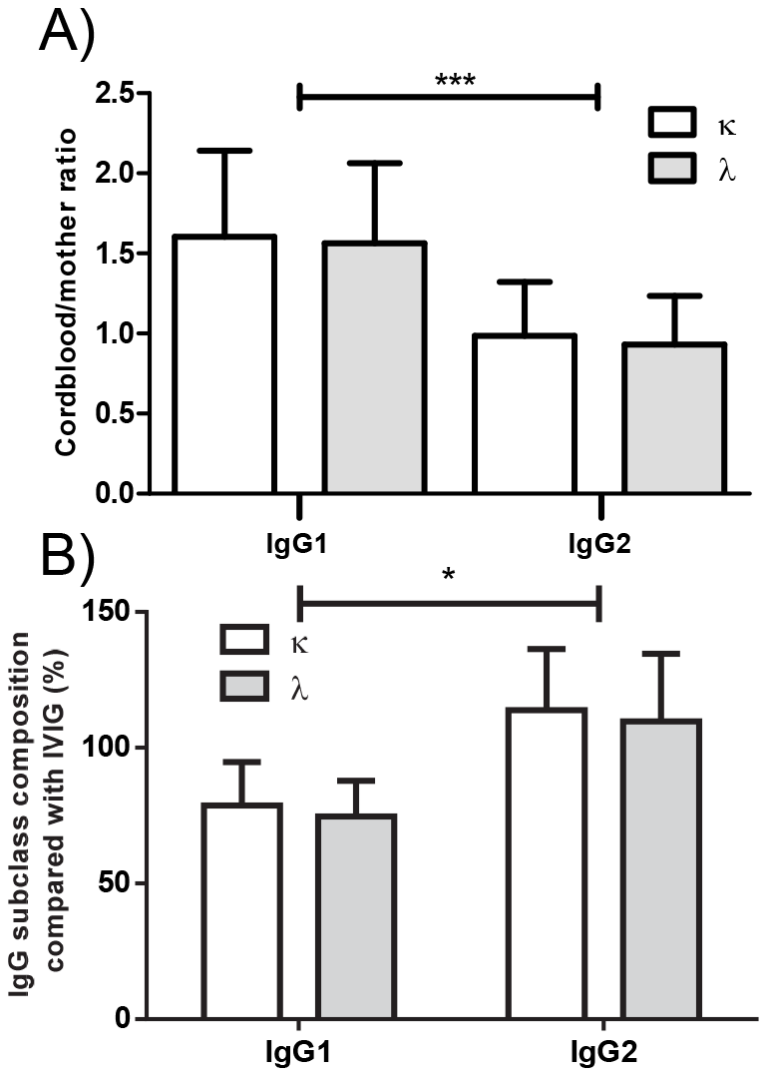
Equal placental transport and serum clearance of IgG1 and 2 light chain isotypes κ and λ. (A) The average IgG1 and IgG2 placental transport (maternal/child) ratios were compared according to their light chain isotype. (B) Clearence of IgG1 and IgG2 κ and λ was investigated in humans by collecting blood from hypogammaglobulinemia patients four weeks after an IVIg transfusion. IgG1 and IgG2 light chain isotypes κ and λ were quantified in serum by subclass- and light chain specific ELISA and subclass composition was compared to that found in the IVIg used. No preferential clearance of one light chain isotype was detectable in either IgG subclass.

As these results are at odds what we expected based on previously published work on a different, but mechanistically similar FcRn-mediated function, describing differences in half-life of IgG2κ and IgG2λ in Balb/C mice, we tested if the half-life of our IgG2 antibodies differed in humans [15]. We analyzed sera from IVIg-treated X-linked agammaglobulinemic patients as we described before, comparing the relative amounts found in the IVIg preparation itself to amounts found in patient serum four weeks later [20]. The relative amount remaining after IVIg-treatment was identical for IgG1κ and IgG1λ, but also for IgG2κ and IgG2λ, indicating that serum half-life does not differ between the light-chain isotypes in humans (Fig. 7B). Curiously, the relative amount remaining after 4 weeks was significantly increased for IgG2 in the IVIg-treated agammaglobulinemic patients (Fig. 7B), while it was significantly decreased for placental transport of IgG2 (Fig. 7A), indicating a fundamental difference between these two processes.

## Discussion

FcRn mediates IgG- transcytosis and recycling, both of which are compromised for IgG3, with reduced serum persistence and placental transport. We recently reported that these effects are attributable to a single amino acid difference between IgG3 and the other subclasses. While IgG1, IgG2 and IgG4 have a histidine at position 435, a key amino acid responsible for the pH-dependent binding to FcRn, most IgG3 allotypes have an arginine at this position, causing them to lose competition for recycling and transcytosis [18], [20]. IgG3 allotypes which express a histidine at this position have equal half-life to that of IgG1, and are transported equally well across the placenta [18], [20].

For IgG2, however, these FcRn-mediated transcytosis and recycling functions seem to diverge [18], [29]. Although IgG1, IgG2 and IgG4 share all known contact residues with FcRn, and FcRn is known to mediate both IgG transcytosis and extend IgG serum persistence in a very similar manner, IgG2 crosses the human placenta with a markedly lower efficiency, with trans-placental transport more closely matching that of IgG3.

Interestingly, Montano and Morrison reported accelerated clearance for IgG2λ, compared to IgG2κ in wild-type mice, indicating that the light chain may affect the interaction of IgG2 with FcRn [15]. This possibility became more plausible after the discovery that IgG2κ uniquely exists in three structurally distinct isoforms, allowing for the possibility that a single isoform of IgG2κ may be influencing the overall interaction of IgG2 with FcRn [4], [5].

We first investigated whether the difference between IgG2κ and IgG2λ could be found in paired mother cord samples. Although the mother/cord ratio was significantly lower for IgG2 than for IgG1 or IgG4, there was no evidence that this was affected by the light chain isotype.

While the original finding that the half-life of IgG2κ and IgG2λ diverge, was observed for mouse-human chimeric antibodies in BALB/c mice, we investigated whether the same applied to fully human recombinant IgG2 antibodies, derived from an human IgG2λ hybridoma but expressed with either κ or λ light chains, and tested them in both C57Bl/6 and BALB-c mice [30], [31]. No differences were found neither in BALB/c or C57Bl/6 mice. Curiously, we found differences in half-lives between experiments. In Balb/C mice we first found on average a half-life of approximately 6.5 days (Fig. 6A), while in another set of experiment it was down to 4.5 days (Fig. 6C). Furthermore, clearance seemed to be accelerated at later time points, as in Fig. 6A. In C57Bl/6 mice, the calculated half-life was again about 6.5 days. One possible reason is that FcRn expression (and consequently, IgG half-life) differs between different mouse strains, but perhaps an even more attractive explanation is that FcRn expression has been reported to be sensitive to NF-κB signalling, suggesting FcRn-levels may also be under the control of the animal's microbial status [32], [33]. This might also explain discrepancy in half-lives of human IgG in mice reported by others [15], [22]. In the absence of FcRn, IgG2 κ and λ also disappeared from circulation at an equal rate.

Since no differences in half-life between recombinant IgG2κ and IgG2λ were observed, we investigated whether a further subdivision of IgG2κ, either the A, the A/B or the B isoform, might be responsible for the observed differences between our results and those previously describing a light chain-dependant half-life extension for IgG2 [15]. As both results were obtained using monoclonal antibodies, the possibility exists that a single isoform constituted the majority of IgG2κ tested, which might skew the results. The serum persistence experiment was executed in both C57Bl/6 (wild-type and FcRn-KO) as well as BALB/c, the mouse strain used by Montano and Morrison [15]. However, no difference between isoforms was observed. Although we did not verify the nature of the chemically A/B isomerization other than by CEX-HPLC, validated previously in [4], we did test the half-life-extension of the light chain isotypes of polyclonal IgG2 obtained from IVIg in agammaglobulinemic patients, and found no indication of preferential rescue of either IgG2κ or IgGλ. As IVIg is an IgG pool from thousands of individuals, and is likely to contain natural ratios of these A/B isomers, this further proofs that IgG2 recycling discriminates between neither the light chain isotypes nor IgG2-isomers. Together with the results showing also no preference for either IgG2κ or IgG2λ during placental transport, this strongly suggests that human FcRn does not discriminate between the two light chain isoforms during intracellular transport.

This leaves the dilemma of explaining the lack of IgG2 transport across the placenta, observed in the paired mother cord samples, compared to equal serum persistence. It has been postulated and subsequently disproved that FcγRIIb found on endothelial cells is involved in IgG transport across the murine yolk sac [34]–[38]. However, Mohanty and co-workers did not fully exclude the possibility that FcγRIIb may play a role in transplacental transport in humans [34]–[37]. If FcγRIIb is indeed involved, it may provide an explanation to the low transport of IgG2, as this is the only subclass with almost no measurable binding affinity to FcγRIIb [39], [40]. However, the group of Ravetch has reported that sialylation of the N-linked glycan at position 297 in the IgG Fc affects binding to mouse FcγR, including mouse FcγRIIb, resulting in 10× lowered binding affinity for both mouse IgG1 and IgG2a [41]. If FcγRIIb is indeed involved in placental transport, this would suggest a skewed transport of sialylated IgG across the placenta. However, we have found no such difference for any of the human subclasses, as all glycoforms of all subclasses were transported equally well across the placenta, suggesting FcRn, which does not require the Fc-glycans for binding, is sufficient for transplacental-IgG transport [42], [43].

While it is conceivable that the requirements for FcRn-binding differ for IgG2 compared to the other subclasses due to the short hinge of IgG2 and the closer proximity of the Fab fragments to the Fc, there is no clear reason why this should affect FcRn-mediated recycling and transcytosis differently. The possibility exists that this extracellular binding also affects FcRn-mediated signaling, as transcytosis and recycling are regulated by different proteins, with actin motor myosin Vb and the GTPase Rab25 initiating transcytosis from the recycling endosome, while Rab11a regulates recycling to the basolateral membrane [44], [45]. The role for the tryptophan-based basolateral-targeting signal identified in the FcRn cytoplasmic tail is also unknown [46]. In support of this view, we did observe that while IgG2 transport through the placenta is indeed low, its recycling and half-life extension in the human circulation is even better than for IgG1, If, and then how, these intracellular mechanisms are differently affected by the stoichiometry of the IgG2 molecules still needs to be elucidated.

In conclusion, we report and confirm that human IgG2 is not efficiently transported across the human placenta, despite a normal half-life. Although a previous report hinted at differential recycling of IgG2 depending on the light chain isotype, we found no preference for either recycling or placental transport of either IgG2κ or IgG2λ. In addition, the half-life extension in mice was not affected by the different IgG2κ structural isoforms. This is suggestive of alternative mechanisms, either involving an alternative receptor or mechanism differentiating between IgG transcytosis and recycling.
